# MTAP deficiency is highly homogeneous in advanced, muscle-invasive urothelial carcinoma of the urinary bladder

**DOI:** 10.1038/s41598-025-10157-0

**Published:** 2025-07-15

**Authors:** Natalia Gorbokon, Katharina Teljuk, Viktor Reiswich, Florian Lutz, Sebastian Dwertmann Rico, Clara Lühr, Christian Bernreuther, Yaryna Klos Al Bunni, Cosima Völkel, Henning Plage, Maximilian Lennartz, Margit Fisch, Sarah Minner, Ronald Simon, Guido Sauter, Henrik Zecha, Stefan Steurer, Thorsten Schlomm, Tatjana Vlajnic, Lukas Bubendorf, Michael Rink, Martina Kluth, Florian Viehweger, Morton Freytag

**Affiliations:** 1 Institute of Pathology, University Medical Center Hamburg-Eppendorf, Martinistr. 52, 20246 Hamburg, Germany; 2 Department of Urology, Charité Berlin, Berlin, Germany; 3 Department of Urology, University Medical Center Hamburg-Eppendorf, Hamburg, Germany; 4 Department of Urology, Albertinen Hospital, Hamburg, Germany; 5 Institute of Pathology, University Hospital Basel, Basel, Switzerland; 6 Department of Urology, Marienkrankenhaus, Hamburg, Germany

**Keywords:** MTAP deficiency, FISH, IHC, Tissue microarray, Urothelial bladder cancer, Tumor heterogeneity, Tumour heterogeneity, Urological cancer, Cancer

## Abstract

Complete expression loss of S-methyl-5′-thioadenosine phosphorylase (*MTAP*) is caused by homozygous 9p21 deletion and results in a critical vulnerability of cancer cells towards drugs targeting multiple different pathways. MTAP deficiency is common in urothelial cancer, but data on its intratumoral heterogeneity—a potential obstacle for targeted therapies—are lacking. To study the heterogeneity of MTAP expression loss and 9p21 deletions in advanced primary urothelial cancers of the urinary bladder, a tissue microarray (TMA) composed of five different tissue spots from different tissue blocks of 105 pT2-4 urothelial carcinomas was analyzed by immunohistochemistry (IHC) and fluorescence in-situ hybridization (FISH). In addition, all tumor containing blocks (1–15, average 6.4) from 41 consecutive pT2-4 carcinomas were analyzed by IHC. Complete absence of MTAP staining (MTAP deficiency) was seen in 34.2% of 385 interpretable TMA samples. All 80 samples with a complete MTAP expression loss and FISH data had a homozygous 9p21 deletion while there were no cases with homozygous deletions within 178 samples with retained MTAP expression (100% FISH/IHC concordance). On a patient level, there was a homogeneous MTAP deficiency in 33%, a heterogeneous MTAP deficiency in 1%, and a retained MTAP expression in 66% of the 98 tumors with at least three interpretable TMA samples. Among 41 consecutive pT2-4 carcinomas from which 1–15 (average 6.4) whole sections were analyzed, MTAP was homogeneously deficient in 34.1%, heterogeneously deficient in 4.9% and homogeneously retained in 61.0%. MTAP deficiency is mostly homogeneous in advanced urothelial carcinoma. MTAP IHC is a near perfect surrogate for the detection of homozygous 9p21 (*MTAP*) deletions. Drugs targeting MTAP deficiency could be highly useful in a relevant subset of invasive urothelial bladder cancers.

## Introduction

The enzyme S-methyl-5′-thioadenosine phosphorylase (MTAP) is essential for the salvage pathway of adenine and methionine synthesis^1^. The *MTAP* gene is located at 9p21.3, immediately adjacent to the *CDKN2A* gene which is homozygously deleted in up to 30% of urothelial carcinomas^2^. Homozygous co-deletions of *MTAP* occur in 80%-90% of *CDKN2A* deleted tumor cells^3^. MTAP deficiency results in a critical vulnerability of cancer cells towards drugs targeting different pathways^4^. Because adenine synthesis can only be maintained by de novo biosynthesis in MTAP deficient cancer cells, inhibition of critical enzymes for folate synthesis terminated MTAP deficient cancer cells in experimental models and also showed in vivo anti-cancer efficiency in urinary bladder cancer patients^4^. MTAP deficient cells can also be eliminated by inhibitors of protein arginine N-methyltransferase 5 (PRMT5) and methionine adenosyltransferase II, alpha** (**MAT2A) (summarized in^5^). PRMT5 is essential for the methylation of numerous proteins and thereby regulates their activity^6^. MAT2A is critical for the synthesis of S-adenosylmethionine (SAM), the substrate of PRMT5^7,8^. As PRMT5 is inhibited by accumulation of the unprocessed MTAP metabolite MTA in tumor cells^9^, MTAP deficiency makes these cells more susceptible to PRMT5 or MAT2A inhibitors (summarized in^5^). Clinical trials with drugs targeting PRMT5, and MAT2A are ongoing^10,11^. Most recently a partial response was reported in 3 and a complete response in one out of 10 MTAP deficient urothelial carcinomas that were previously treated by multiple lines of chemotherapy after single agent treatment by the MAT2A inhibitor IDE397^12^.

Tumors that can benefit from targeting MTAP deficiency must be identified by molecular tumor analysis. For this purpose, immunohistochemistry (IHC) is a suitable method, because MTAP is ubiquitously expressed in normal cells^13^. In a recent study on 1,792 analyzable muscle-invasive urothelial carcinomas, we found MTAP deficiency in 24.0% of cases and observed a sensitivity of 98.4% and a specificity of 99.6% for MTAP IHC to detect homozygous 9p21 deletions^14^. It is a conceptual weakness of biomarker testing in tumor biopsies, however, that the biomarker status is always determined on tumor tissues removed from the patient, while the treatment should target other fractions of the tumor which have remained in the patient. A different MTAP expression status in metastatic tumor fractions as compared to the molecularly analyzed primary tumor tissue could either prevent response to therapy or—in case of a change from MTAP positivity to MTAP deficiency—lead to a situation where a treatable cancer would not be detected by standard diagnostic procedures. Studies analyzing the extent of heterogeneity of biomarker expression in cancer – including also advanced urothelial bladder carcinomas—have shown that the level of heterogeneity greatly depends on both the biomarker and the tumor type^15–25^.

As data on the potential heterogeneity of MTAP expression in muscle-invasive urothelial carcinomas are lacking, we analyzed all available tumor containing tissue blocks from 41 consecutive primary pT2-4 cancers, and also utilized a “urothelial bladder cancer heterogeneity tissue microarray (TMA) containing five different samples from five different tumor blocks of 105 further pT2-4 cancers.

## Materials and methods

### Tissue microarray (TMA)

The urothelial bladder cancer heterogeneity TMA was constructed from the cancers of 105 patients who underwent either transurethral resection or cystectomy for muscle-invasive urothelial carcinoma of the urinary bladder (pT2-4) and from which the tissue had been analyzed at the Institute of Pathology of the University Medical Center Hamburg-Eppendorf. The selection criteria included the availability of at least five different cancer containing tissue blocks per patient. Tissues were fixed in 4% buffered formalin and then embedded in paraffin. For TMA construction, one single 0.6 mm tissue core was taken from each tumor containing block, resulting in a tissue microarray with a total of 525 tissue cores. The use of archived remnants of diagnostic tissues for manufacturing of TMAs and their analysis for research purposes as well as patient data analysis has been approved by local laws (HmbKHG, §12) and by the local ethics committee (Ethics commission Hamburg, WF-049/09). All work has been carried out in compliance with the Helsinki Declaration.

### Whole section heterogeneity analysis

To search for areas with discrepant/heterogenous MTAP status, all available tissue blocks of 41 consecutive primary pT2-4 urothelial bladder carcinomas were subjected to IHC analysis. This analysis involved a total of 261 whole sections from 41 cancers (average: 6.4 slides per patient; minimum: 1, maximum: 15).

### Immunohistochemistry (IHC)

IHC was executed as previously described^14^. Freshly prepared 2.5 µm TMA or whole sections (slide age < 1 week) were analyzed in a Dako Omnis automated stainer (Agilent Technologies, Santa Clara, CA, USA) using the EnVision FLEX, High pH Kit (Agilent Technologies, Santa Clara, CA, USA, #GV800). Slides were deparaffinized with Clearify™ agent (Agilent Technologies, Santa Clara, CA, USA, #GC810) and exposed to heat-induced antigen retrieval for 30 min at 97 °C in Target Retrieval Solution, High pH reagent (9.0; part of Agilent kit #GV800). Primary antibody specific for MTAP (recombinant rabbit monoclonal, MSVA-741R, MS Validated Antibodies GmbH, Hamburg, Germany, #5293-741R) was applied at ambient temperature for 30 min at a dilution of 1:50. Endogenous peroxidase activity was blocked with Peroxidase-Blocking-Reagent (part of Agilent kit #GV800) for 3 min. Bound antibody was visualized using the EnVision FLEX, High pH kit reagents DAB + Chromogen and Substrate Buffer (parts of Agilent kit #GV800) and EnVision FLEX + Rabbit LINKER (Agilent Technologies, Santa Clara, CA, USA; #GV809) according to the manufacturer’s directions. The sections were counterstained with hemalaun. For tumor tissues in TMA and whole section analysis, the average staining intensity of unequivocally neoplastic cells was estimated as 0, 1 + , 2 + , and 3 + . For the classification of a tumor as completely negative (0), presence of unequivocal MTAP staining in tumor adjacent normal tissue was required. Tumors with complete absence of MTAP staining in cancer cells and a lack of stromal cells with unequivocal MTAP staining were considered “non-informative”. Tumors with a staining intensity of 1 + , 2 + , and 3 + were grouped as “MTAP retained” tumors. Heterogeneity was determined as the presence of MTAP deficient and MTAP retained areas in the tissue of one tumor spot, one whole section, or in two or more tissue spots /whole sections of one tumor.

### Fluorescence in-situ hybridization (FISH)

FISH was executed as previously described^13^. In brief, five micrometer TMA sections were deparaffinized, rehydrated and exposed to heat-induced denaturation for 10 min in a water bath at 99 °C in P1 pretreatment solution (Agilent Technologies, Santa Clara, CA, USA; #K5799). For proteolytic treatment, slides were added to VP2000 protease buffer (Abbott, Chicago, IL, USA; #2 J.0730) for 200 min at 37 °C in a water bath. A commercial FISH probe kit containing both, a 9p21 probe with a size of 315 kb including both the *CDKN2A* and the *MTAP* gene—and a centromere 9 probe were utilized for 9p21 copy number detection (ZytoLight^®^ SPEC CDKN2A/CEN 9 Dual Color Probe, Zytovision, Bremerhaven, Germany; #Z-2063). Hybridization was performed overnight at 37 °C. Posthybridization washes were done according to the manufacturer’s direction at 37 °C. Stained tissues were manually interpreted and copy numbers of 9p21 and centromere 9 were estimated for each tissue spot. Presence of roughly equal numbers of 9p21 and centromere 9 signals in tumor cell nuclei was considered as 9p21 normal. Presence of fewer 9p21 signals than centromere 9 probe signals in at least 60% tumor cell nuclei or one 9p21 and one centromere 9 signal (monosomy of chromosome 9) in nearly all tumor cell nuclei were considered as heterozygous deletion. Complete absence of 9p21 signals but presence of centromere 9 signals in the tumor cell nuclei and of unequivocal 9p21 signals in tumor adjacent normal cell nuclei, was considered a homozygous 9p21 deletion. Tissue spots lacking 9p21 signals in all (tumor and normal cell) nuclei were excluded from analysis.

### Statistics

Statistical calculations were performed with JMP18^®^ software (SAS^®^, Cary, NC, USA). Contingency tables and the chi^2^-test were performed to search for associations between MTAP immunostaining and 9p21 status.

## Results

### Technical results

TMA spots were informative for MTAP IHC in 385 (73.3%) and for MTAP FISH in 258 (49.1%) of the 525 arrayed tumor samples. The remaining 140/267 tissue samples were not interpretable because of a lack of unequivocal tumor cells, absence of MTAP IHC staining in both cancerous and normal tissues, absence of interpretable FISH signals, or a lack of the entire tissue spot in the TMA section.

### MTAP FISH vs. IHC results

**Fig. 1:**
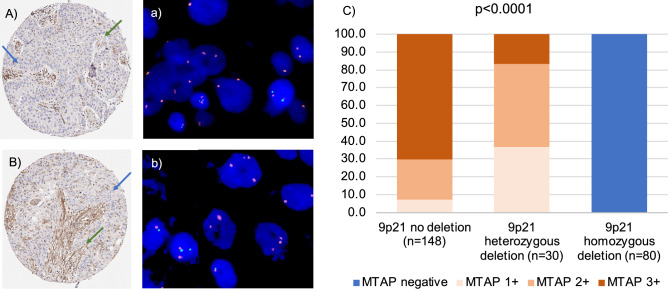
MTAP immunostaining and 9p21 copy number status. (**A**,**B**) show tumor areas with MTAP deficiency (intensity “0”, blue arrow) and normal tissue with positive MTAP staining as control (intensity “2”, green arrow). (**a**) and (**b**) show tumor cell nuclei with homozygous MTAP deletion without any green 9p21 signals and presence of orange chromosome 9 signals in the tumor cell nuclei and 9p21 and chromosome 9 signals in adjacent normal cell nuclei. (**c**) shows the comparison of MTAP immunostaining and 9p21 copy number status.

Complete absence of MTAP staining was seen in 132 (34.2%) of 385 IHC interpretable TMA samples while 31 (8.1%) showed 1 + , 69 (17.9%) 2 + , and 153 (39.8%) 3 + MTAP staining. There was a strong correlation between IHC and FISH data (p < 0.0001, Fig. 1). All 80 samples with a complete MTAP expression loss and FISH data of the 9p21 region had a homozygous 9p21 deletion while there were no cases with homozygous deletions within 178 samples with retained MTAP expression (100% concordance). Heterozygous deletions were seen in 11 (50%) of 22 samples with 1 + staining but in only 14 (29.8%) of 47 with 2 + and 5 (4.6%) of 109 with 3 + positivity by IHC (p < 0.0001).

### MTAP heterogeneity within individual patients (TMA)

**Fig. 2:**
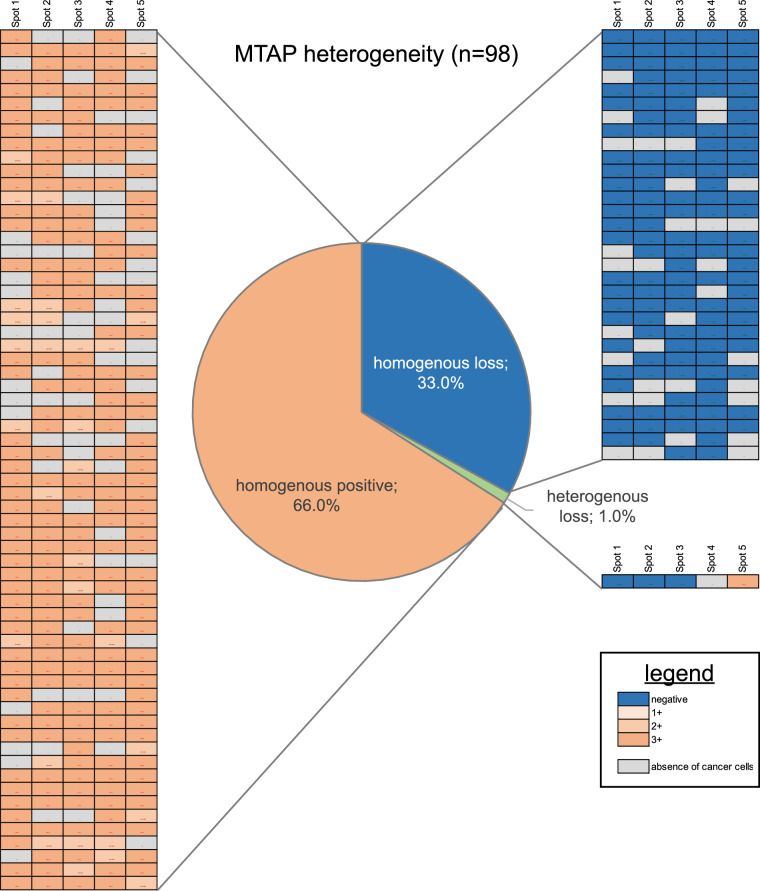
Heterogeneity of MTAP immunostaining.

**Fig. 3:**
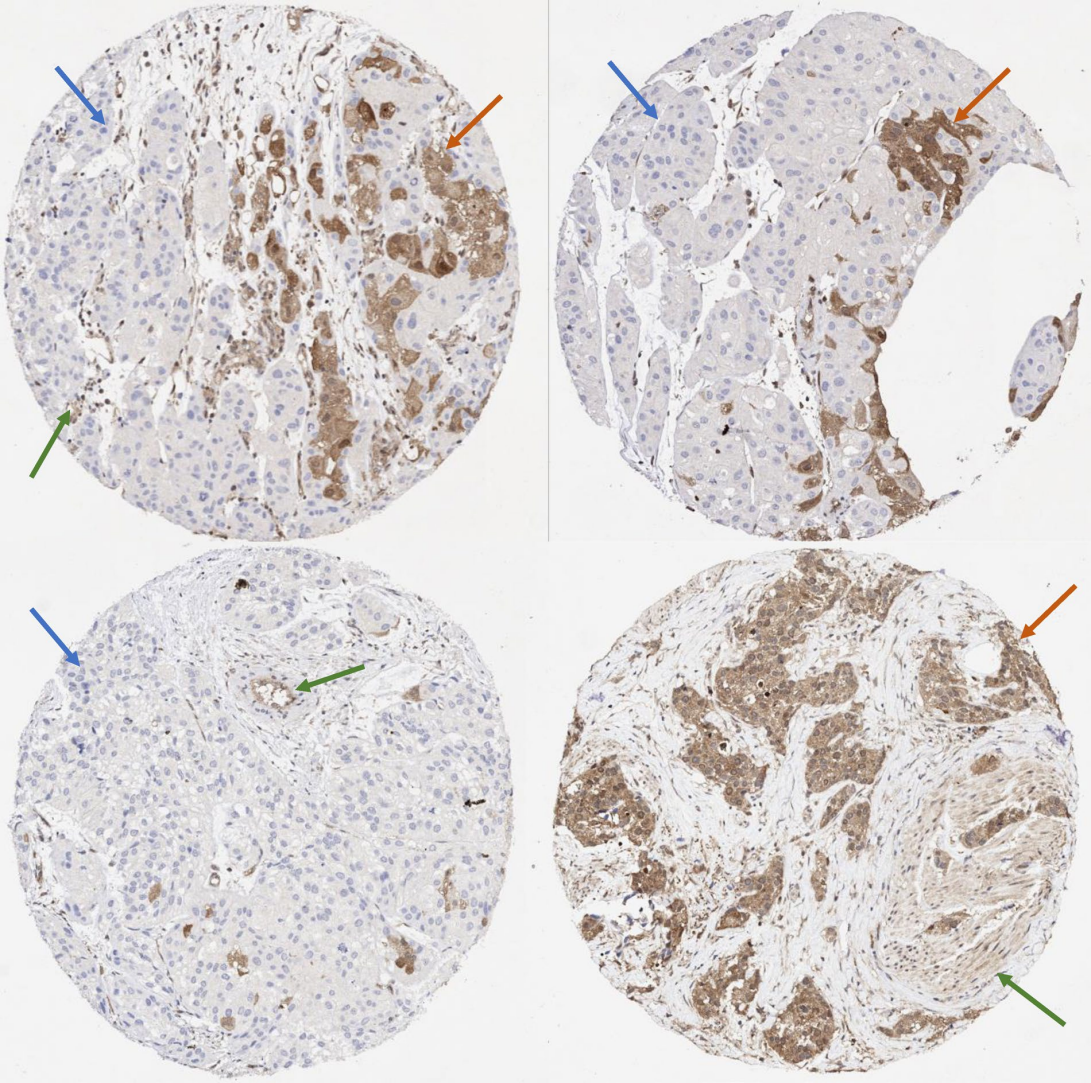
Heterogenous MTAP immunostaining in four tumor samples of one urothelial bladder carcinoma. Images show tumor areas with MTAP deficiency (intensity “0”, blue arrow) and MTAP retained expression (intensity “2”, red arrow) as well as normal tissue with positive MTAP staining as control (intensity “2”, green arrow).

On a patient level, there was a homogeneous MTAP deficiency in 33%, a heterogenous MTAP deficiency in 1% and a retained MTAP expression in 66% of 98 tumors with at least three interpretable TMA samples (Fig. 2). Images from the MTAP heterogeneous tumor are shown in Fig. 3.

### Whole section analysis

**Fig. 4:**
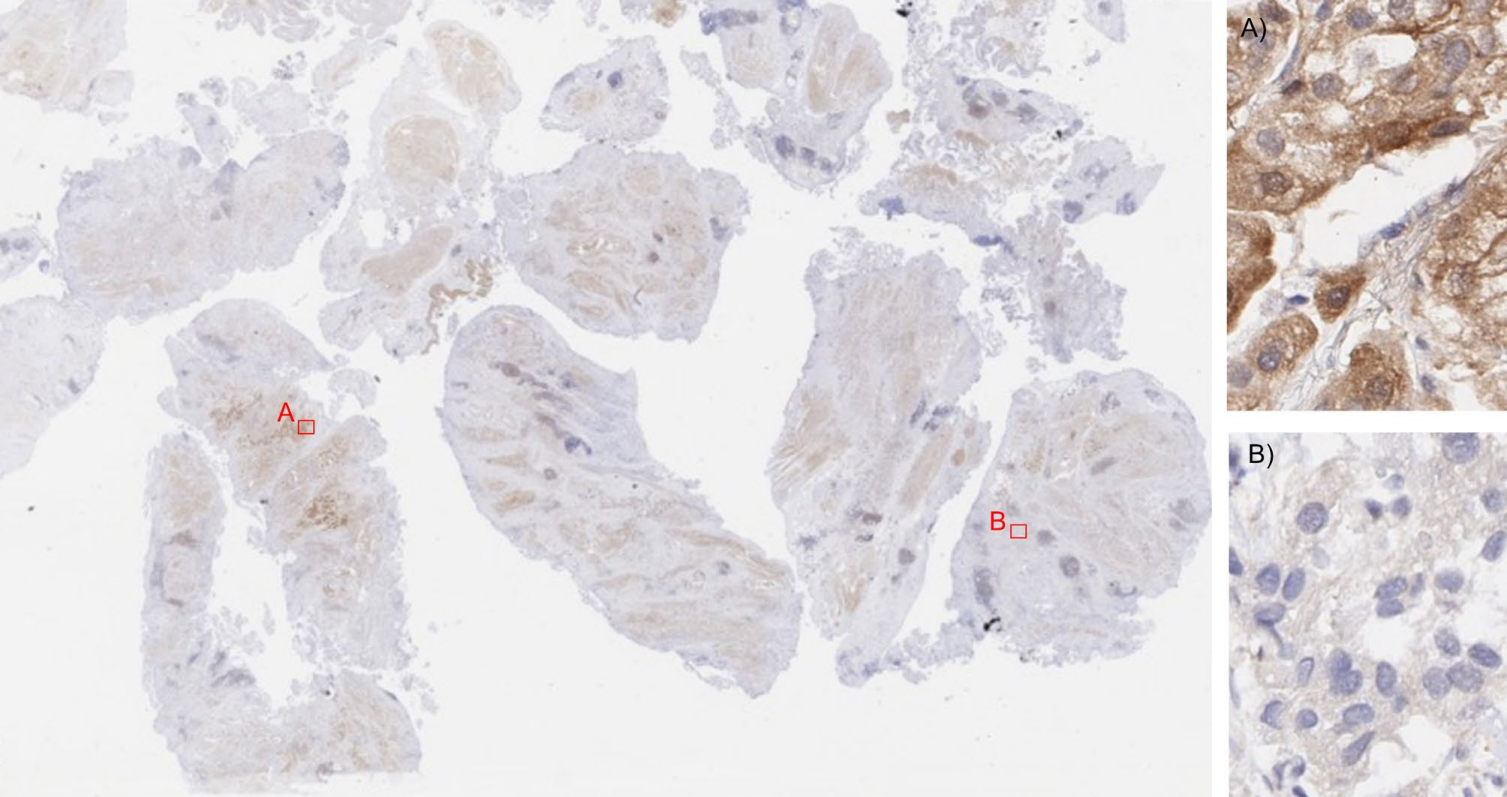
Example of heterogenous MTAP immunostaining in one whole section urothelial bladder carcinoma. (**A**) positive MTAP immunostaining and (**B**) negative MTAP immunostaining.

Among 41 consecutive primary pT2-4 urothelial bladder carcinomas from which 1–15 (average 6.4) whole sections were analyzed, MTAP was homogeneously deficient in 34.1%, heterogeneously deficient in 4.9% and homogeneously retained in 61.0%. The complete data from our heterogeneity analysis is given in Table 1. An example of an MTAP heterogeneous cancer is given in Fig. 4.

**Table 1:** MTAP immunostaining in whole section analysis of 41 urothelial bladder carcinomas.

Patient ID	Tumor stage	Examined tumor sections	MTAP immunostaining in each tumor section														
			1	2	3	4	5	6	7	8	9	10	11	12	13	14	15
1	pT2-4	9	Negative	Negative	Negative	Negative	Negative	Negative	Negative	Negative	Negative						
2	pT2-4	1	Positive														
3	pT2-4	10	Positive	Positive	Positive	Positive	Positive	Positive	Positive	Positive	Positive	Positive					
4	pT2-4	4	Positive	Positive	Positive	Positive											
5	pT2-4	10	Heterogenous negative	Heterogenous negative	Heterogenous negative	Heterogenous negative	Heterogenous negative	Heterogenous negative	Negative	Negative	Negative	Negative					
6	pT2-4	5	Negative	Negative	Negative	Negative	Negative										
7	pT2-4	10	Positive	Positive	Positive	Positive	Positive	Positive	Positive	Positive	Positive	Positive					
8	pT2-4	4	Positive	Positive	Positive	Positive											
9	pT2-4	6	Positive	Positive	Positive	Positive	Positive	Positive									
10	pT2-4	2	Negative	Negative													
11	pT2-4	2	Positive	Positive													
12	pT2-4	3	Heterogenous negative	Heterogenous negative	Heterogenous negative												
13	pT2-4	6	Positive	Positive	Positive	Positive	Positive	Positive									
14	pT2-4	10	Positive	Positive	Positive	Positive	Positive	Positive	Positive	Positive	Positive	Positive					
15	pT2-4	10	Positive	Positive	Positive	Positive	Positive	Positive	Positive	Positive	Positive	Positive					
16	pT2-4	2	Negative	Negative													
17	pT2-4	5	Positive	Positive	Positive	Positive	Positive										
18	pT2-4	3	Positive	Positive	Positive												
19	pT2-4	10	Positive	Positive	Positive	Positive	Positive	Positive	Positive	Positive	Positive	Positive					
20	pT2-4	6	Positive	Positive	Positive	Positive	Positive	Positive									
21	pT2-4	11	Positive	Positive	Positive	Positive	Positive	Positive	Positive	Positive	Positive	Positive	Positive				
22	pT2-4	15	Positive	Positive	Positive	Positive	Positive	Positive	Positive	Positive	Positive	Positive	Positive	Positive	Positive	Positive	Positive
23	pT3a	11	Positive	Positive	Positive	Positive	Positive	Positive	Positive	Positive	Positive	Positive	Positive				
24	pT3b	10	Positive	Positive	Positive	Positive	Positive	Positive	Positive	Positive	Positive	Positive					
25	pT2-4	9	Positive	Positive	Positive	Positive	Positive	Positive	Positive	Positive	Positive						
26	pT2-4	8	Negative	Negative	Negative	Negative	Negative	Negative	Negative	Negative							
27	pT2-4	10	Positive	Positive	Positive	Positive	Positive	Positive	Positive	Positive	Positive	Positive					
28	pT1	2	Positive	Positive													
29	pT2-4	7	Negative	Negative	Negative	Negative	Negative	Negative	Negative								
30	pT2-4	2	Negative	Negative													
31	pT2-4	4	Negative	Negative	Negative	Negative											
32	pT2-4	2	Negative	Negative													
33	pT2-4	4	Negative	Negative	Negative	Negative											
34	pT2-4	3	Negative	Negative	Negative												
35	pT4a	6	Negative	Negative	Negative	Negative	Negative	Negative									
36	pT2-4	2	Negative	Negative													
37	pT2-4	10	Positive	Positive	Positive	Positive	Positive	Positive	Positive	Positive	Positive	Positive					
38	pT2-4	10	Negative	Negative	Negative	Negative	Negative	Negative	Negative	Negative	Negative	Negative					
39	pT3a	5	Positive	Positive	Positive	Positive	Positive										
40	pT2-4	10	Positive	Positive	Positive	Positive	Positive	Positive	Positive	Positive	Positive	Positive					
41	pT2-4	2	Negative	Negative

## Discussion

That analyses of molecular heterogeneity in tumors are only rarely done is obviously due to the inherent challenges of such analyses, especially in advanced carcinomas presenting with large tumor masses. In case of a tumor measuring 5 cm in diameter, the analysis of one 4 µm whole section containing 3 × 2 cm of cancer tissues would only enable the analysis of only approximately 1/272,500 of the entire tumor mass all of which located in one contiguous tumor area. To cost-efficiently analyze in many different tumors several different tumor areas which are as distant from each other as possible, we constructed a heterogeneity TMA containing five cancer samples per patient that were all taken from different tumor containing tissue blocks. This approach enabled a rather comprehensive heterogeneity analysis of 105 bladder cancer patients by staining only five TMA sections. This “heterogeneity TMA” approach had previously been successfully used for assessing the heterogeneity of genetic alterations of *PTEN* and *TMPRSS2:ERG* fusion in prostate cancer^19^, gene amplification in gastric cancer^22^, p53 and HER2 expression in colorectal cancer^26^, and EGFR copy number gain in non-small cell lung cancer^27^.

Our data demonstrate a high homogeneity of MTAP deficiency in advanced urothelial carcinomas of the urinary bladder. Only 1.0% of 98 cancers in our heterogeneity TMA study and only 2 (4.9%) of 41 cancers in our “whole primary tumor analysis” showed a heterogeneous MTAP finding. These data suggest that MTAP deficiency in advanced invasive urothelial carcinomas is among the most homogeneous molecular alterations that can be found in cancer. However, results from previous studies had demonstrated that the rate of heterogeneous cases varies markedly between individual molecular markers and between tumor entities. While high homogeneity rates had been found for loss of mismatch repair genes across several tumor entities including colorectal neuroendocrine carcinomas^18^, ovarian^16^, pancreatic^15^, and prostate cancer^17^, heterogeneity for *HER2* amplification depends on the cancer type. For example, heterogeneity of high-level *HER2* amplification and overexpression has been found to be minimal (up to 11%) in breast cancer^21^, moderate (up to 50%) in stomach cancer^22^, and high (> 60%) in urothelial carcinoma^20^. A high rate of heterogeneity was, for example, also found for *ALK* rearrangements in lung cancer^24^, *PTEN* deletion in prostate cancer^19^, and *BRAF* mutation in lung adenocarcinoma^23^.

The results of this study identified 33–39% of our muscle-invasive urothelial carcinomas of the urinary bladder as MTAP deficient. This number is slightly higher than the 24% in our previous study analyzing 1,792 pT2-4 carcinomas by using an identical protocol and a similar approach for evaluation. Given the highly homogeneous nature of MTAP deficiency, this difference cannot be explained by the higher number of samples (n = 5) that were analyzed per tumor in our “heterogeneity TMA” or the whole section approach in 41 cases. Considering the significant increase of MTAP deficiency from pT2 (22.2%) to pT3 (30.0%) and pT4 (31.2%) carcinomas in our earlier study^14^, we would speculate that the rather high rate of MTAP deficient cancers in our present series is caused by a selection of large tumors with availability of as many as possible tumor containing tissue blocks for constructing a heterogeneity TMA and potentially also by the relatively low number of cases (n = 41) in our consecutive series. All together these data suggest considerable utility of drugs targeting MTAP deficiency in advanced urothelial carcinoma. Due to the common co-deletion of *MTAP* and *CDKN2A* combined PRMT5 and CDK4/6 inhibitor therapies could also be considered^28^. Of note, experiments on cell lines have suggested that adenine uptake from the tumor environment into the tumor cell may limited treatment success^29^.

It is of note that a highly validated IHC assay has been applied in this study. The antibody was validated by a comparison with RNA data and with a second independent antibody according to the recommendations of the International Working Group for Antibody Validation (IWGAV)^13^. The staining protocol had been titrated on a TMA containing urothelial bladder carcinomas with 9p21 wild type (n = 20), heterozygous deletions (n = 20), and homozygous deletions (n = 20) to obtain maximal staining in non-neoplastic cells while background staining was still lacking in homozygously deleted cancer cells^14^. Accordingly, the concordance between IHC and FISH data was near perfect (100%) in this study and even better than in our previous report (98.4%). We are certain that this “improvement” of our concordance rate is due to the use of consecutive tissue section which enabled a case-by-case review of potentially discordant findings. In several instances this approach enabled the identification of homozygous 9p21 deletions in patients where minute cancer cell populations had initially remained undetected in our FISH analysis. These findings underscore the high utility of IHC for rapid and inexpensive detection of MTAP deficiency as well as the assumption that homozygous 9p21 deletion are the main mechanism of MTAP deficiency in urothelial carcinoma of the bladder.

Our data are somewhat in contrast with those from Vanijic et. al.^30^ who had described heterogeneity of MTAP deficiency in 41 of 164 (25%) of cases by IHC in a whole section analysis. While these authors emphasized that most heterogeneous cases involved pTa tumors and flat urothelium showing an intermixture of MTAP deficient tumor cells with MTAP positive urothelial cells that could not unequivocally be identified as neoplastic they did not mention whether and at what frequency heterogeneous pT2-4 cancers were also seen. As we are using MTAP IHC in our routine work-up of newly diagnosed urothelial neoplasms we do regularly see such MTAP status heterogeneity in dysplastic flat lesions and in pTa tumors. Based on the available data, it appears possible that molecular cancer progression towards homozygous 9p21 deletion—potentially via heterozygous deletions—mainly occurs during the non-invasive phase of tumor progression while the final MTAP status may be largely determined at the time when the cancer has progressed into invasive disease. Given the homogeneity of MTAP deficiency in most advanced primary tumors, MTAP deficiency should also be retained in their metastases although metastases were not analyzed in our study. In agreement with the concept of an increased rate of polyclonality of non-invasive urothelial neoplasms, studies analyzing p53 alterations in bladder cancer have found multiple different mutations and non-mutated neoplastic clones in one tumor foci of many non-invasive neoplasms while only one of these neoplastic cloned managed to become invasive eventually^31^.

In summary, our data demonstrate that MTAP deficiency is frequent and highly homogeneous in advanced muscle-invasive urothelial carcinoma of the urinary bladder. Advanced urothelial carcinoma may thus represent an ideal cancer type for new drugs targeting MTAP deficient cancer cells.

## Data Availability

The datasets used and/or analyzed during the current study are available from corresponding author on reasonable request.

## Abbreviations

FISH: Fluorescence in-situ hybridization
IHC: Immunohistochemistry
MAT2A: Methionine adenosyltransferase II, alpha
MTAP: S-methyl-5′-thioadenosine phosphorylase
PRMT5: Protein arginine N-methyltransferase 5
SAM: S-adenosylmethionine
TMA: Tissue microarray
